# Development of an Implantable Wireless and Batteryless Bladder Pressure Monitor System for Lower Urinary Tract Dysfunction

**DOI:** 10.1109/JTEHM.2019.2943170

**Published:** 2019-10-14

**Authors:** Yihua Zhong, Bolin Qian, Yaguang Zhu, Zhaohui Ren, Junming Deng, Jinghua Liu, Qianrui Bai, Xu Zhang

**Affiliations:** 1 School of Biomedical EngineeringCapital Medical University 12517 Beijing100069China; 2 Beijing Key Laboratory of Fundamental Research on Biomechanics in Clinical ApplicationCapital Medical University 12517 Beijing100069China; 3 Jiangsu MOCOTO Medical Technology Company, Ltd.Suzhou215123China; 4 Covidien Medical Devices Technology Company, Ltd.Shanghai200070China

**Keywords:** Long-term implantation, real-time bladder pressure measurement, sensor, wireless power transmission

## Abstract

Background: Closed-loop neuromodulation based on bladder pressure is an effective therapy for lower urinary tract dysfunction. The catheter-based cystometry normally used for bladder pressure measurement is not conducive to patient health because it will bring great mental stress to the patient and increase the risk of infection. Method: This paper designs and implements an implantable wireless and batteryless bladder pressure monitor system that monitors bladder storage in real time by implanting a miniature packaged sensor which transmits the feedback signal to the external receiver through BLE (Bluetooth Low Energy). The implanted part is powered by a dedicated magnetic resonance based wireless power transmission system, which means no battery is needed. Results: The maximum distance to which power can be transmitted is 7cm. The in vitro experiment proves that the system performance can meet the requirement of bladder pressure monitoring. The animal experiment uses rabbits as a model to verify the effectiveness of the system. After implantation, this system can work for a long time without replacing the battery. Conclusion: This system can monitor the pressure of the bladder and provide a basis for Closed-loop neuromodulation in patients with lower urinary tract dysfunction.

## Graphical abstract

This is an implantable wireless and batteryless bladder pressure monitor. It can monitor the pressure of the bladder at any time and provide a basis for Closed-loop neuromodulation in patients with lower urinary tract dysfunction.

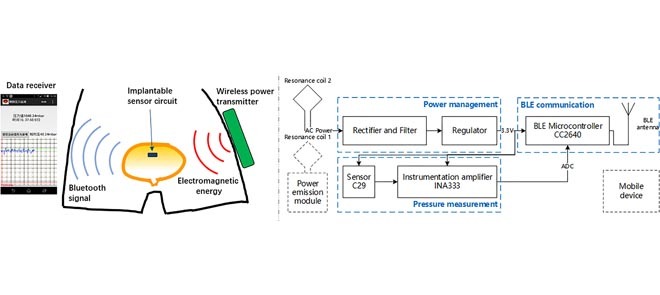

## I. Introduction

Neurogenic bladder is a type of disease that causes lower urinary tract dysfunction (i.e., urine storage and urination dysfunction) due to nervous system disease. It will result in a series of symptoms like urinary incontinence, urinary retention and frequent micturition. Lower urinary tract dysfunction causes great inconvenience to the patient, and the renal failure caused by it will threaten the patient’s life if it can’t be controlled. It is worth noting that its treatment is still a medical problem that hasn’t been solved yet.

An effective method for alleviating the symptoms of bladder dysfunction is to use an implantable nerve stimulator to stimulate the nerves that govern the function of bladder and restore the lower urinary tract function. However, most clinically applied implantable nerve stimulators are open-loop systems [1]. They don’t have the sensing or measuring function for bladder reservoir volume. Usually the stimulators’ duty cycle can only be set by user experience, which is incorrect. Therefore, using a closed-loop feedback bladder function regulation system that can detect the pressure of the bladder in real time and compare it with the voiding threshold value is the best solution to control the bladder activity [2].

In clinical practice real-time pressure signal measurements are mostly invasive, such as measuring bladder pressure by a urodynamic instrument. Although the current catheter has been greatly improved in the sensor and package, the disadvantages of catheter manometry still exist, and the frequent use of the catheter will bring great mental stress to the patient and increase the risk of infection [3]. By implanting a bladder pressure monitoring micro-sensor system that can communicate with the outside to perform real-time bladder pressure measurement can avoid the drawbacks of frequent measurement and guide the more effective work of the neurological function control device.

This kind of ideal implantable bladder pressure detection circuit should be a wireless long-term device which can be implanted into the body through minimally invasive surgery. Recent years, many research groups have been conducting research in this field. Some sensing systems powered by micro-batteries are designed to realize real-time monitoring bladder pressure, but these systems cannot work for a long time because of the tradeoff between the size limitation for implantation and the battery size [4]–[6]. Majerus.S from Case Western Reserve University improved the power supply mode. In his research, a micro-battery which can be recharged with Radio Frequency is used to power the implant part. In this way, the implanted system can work in a long time [7]–[9]. He conducted the sensor module implantation within the bladder wall of large animal models, where can protect the sensor from urine corrosion. However, mucosal erosion occurred 2–4 weeks after implantation leading to device expulsion during urination [10]–[13]. The latest research is Aaron D. Mickie’s research [14] in 2019. He developed a soft, high-precision biophysical sensor system that allows continuous measurements of bladder volume. This well-designed soft strain gauge yields real-time information on bladder function in a rat model. Although the experiment has achieved good results, due to the different bladder shapes of different animals, even the same animal has great differences between individuals, and there is still a long way to go to reflect the amount of bladder urine through the bladder deformation.

**FIGURE 1.**
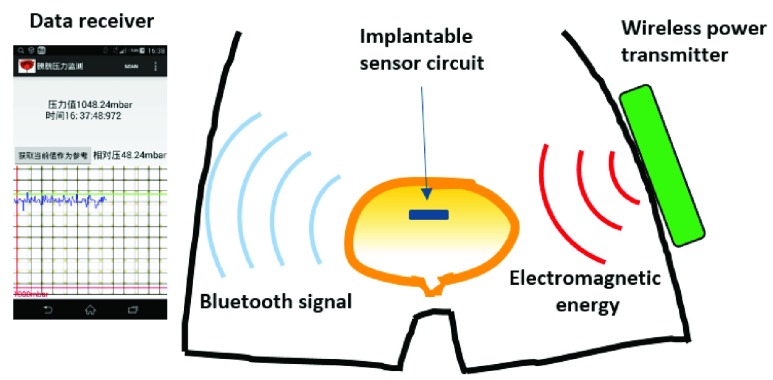
The operating diagram of the bladder pressure monitor system.

Here, we present a new bladder pressure monitoring system design that includes a small low-power implantable sensor circuit. The sensor module attempts to implement both wireless and batteryless modes of operation. It can be powered by a wireless power transmitter using magnetic resonance based wireless energy transfer technology. This power mode prevents the adverse effects of the battery on weight and space. It measures bladder pressure in real time after implantation into the bladder cavity and transmits the data to an external receiver via BLE (Bluetooth Low Energy). The receiver is an Android mobile device that displays pressure data in a waveform graph via a customized APP (see Fig. 1).

## II. Methods and Procedures

### A. Wireless Implantable Sensor Module Design

To achieve the small size necessary for a long-term implantation, medical devices require ultra-low power consumption and fewer components. For this purpose, we use lower power chips and more integrated components. To maintain a small power budget while providing real-time transmission of the pressure data, we choose BLE technology to realize wireless communication. Compared with some other wireless communication methods like sub-1GHz RF and ZigBee, BLE has lower power consumption and a higher data transfer rate [15]. On the other hand, the magnetic coupling resonance wireless energy transmission technique is adopted for providing power to the implant module. Compared with the traditional transcutaneous coupling technique, this technique can achieve higher power and a longer distance [16]. It is suitable for providing power to deep implanted medical devices such as the bladder pressure sensor that will be implanted deep inside the abdomen. The implant module can run with enough electrical energy with this method and attain a smaller size after the batteries are removed.

**FIGURE 2.**
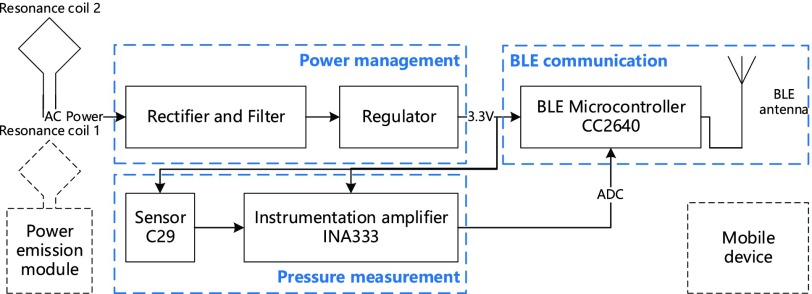
The block diagram of the implantable bladder pressure sensor module.

The circuit block diagram of the implantable bladder pressure sensor module is shown in Fig. 2. The wireless implant module has three main components: a pressure measurement circuit, a power management circuit and a BLE communication circuit. This entire module is controlled by the CC2640 BLE Microcontroller chip (TI) to collect the pressure value which comes from the sensor circuit and transmit them through the Bluetooth antenna. The external power emission module sends power to the implanted part through a pair of resonance coils.

#### 1) Pressure Measurement Circuit Design

In the design of the pressure measurement circuit, C29 (TDK) liquid pressure sensor is served as the analog front end. The pressure signal is amplified by the INA333 Instrument Amplifier (TI) to meet the requirement of bladder pressure measurement. This analog value is then converted to digital value by the A/D converter integrated inside the CC2640 microcontroller.

Normally, the bladder pressure range is 35cm H_2_*O* – 100cm H_2_O, but this number can be larger for patients with lower urinary tract dysfunction. To measure the liquid pressure in the bladder of the patient accurately, the measurement range should fall within 0–200cm H_2_O and the measurement resolution of the sensor should be at least 1cm H_2_O. C29 is a high precision hydraulic sensor with a size of 2.2~2.7mm. Its pressure measurement range is 0–3bar (absolute pressure) which meets the requirement, and the sensitivity is 0.055mV/cm H_2_O when it is powered by the 3.3V voltage supply [17]. To capture the small pressure signals that come from the sensor with the integrated 12-bit ADC, an instrument amplifier should be used to amplify the signals properly. Here, we use INA333 to amplify the signals 46.5 times; the sensitivity becomes 2.555mV/cm H_2_O. For the on-chip stable 4.3V reference voltage, the 12-bit ADC is able to capture signals above 1mV, so the analog signals can be converted into digital values successfully; the theoretical calculation value of measurement resolution is about 0.4cmH_2_O, which meets the requirement.

#### 2) Power Management Circuit Design

The power management circuit consists of a rectifier, a filter circuit and an ultra-low power voltage regulator. The energy receiving resonant coil induces AC from the alternating electromagnetic field. This electrical energy can be utilized by the implant module as DC after rectification and filtering. Considering the small volume, we choose the half-wave rectifier capacitance filter circuits. The sensor and the ADC need a very stable voltage source, so the voltage regulator chip is used to output stable 3.3V voltage as the working voltage of the entire implantable sensor circuit.

#### 3) BLE Communication Circuit Design

Although the BLE technology can transmit a large amount of data in a short period of time, we just send data every 2 seconds to reduce the energy consumption as the bladder pressure is a slow changing signal. In this design, the sampling frequency of the ADC is set to 5Hz and the ten sets of data collected will be averaged before transmission. We also use CC2640’s unique sensor controller which can control the peripherals independently of the main CPU to save more current consumption.

**FIGURE 3.**
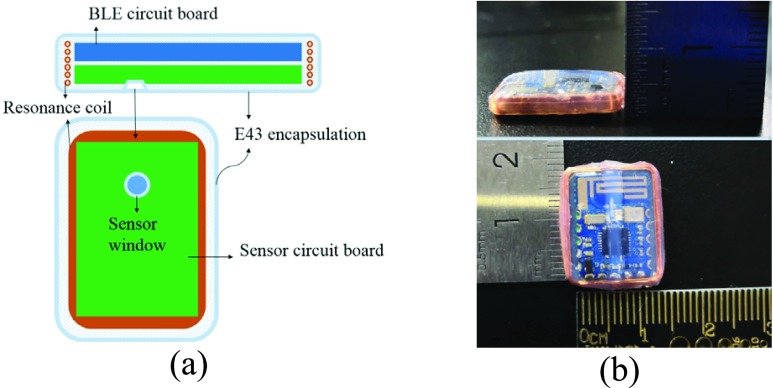
The encapsulated bladder pressure monitor. (a) Package diagram; (b) Size diagram.

To ensure the safety of long-term implantation, the implant module is fabricated by Wacker E43 silicone rubber material [18] (see Fig. 3a). One side of the implant module is the BLE circuit board and the other side is the sensor circuit board. Fig. 3b shows that the actual size after encapsulation is 17*13*3mm. Bench tests will be conducted first before doing acute or chronic implant studies.

### B. Resonant Coil Design

The basic principle of the magnetic coupling resonance wireless energy transmission method is that two coils with the same resonant frequency can enhance the efficiency of electrical energy transfer. High quality factor resonators can transfer energy more efficiently at lower coupling rates and have little influence on other non-resonant objects in the environment [19]. Compared with a traditional wireless energy transmission method, the new method can achieve further transmission distance and can also provide an angle offset tolerance [20]. The design of the resonators is very important for this wireless energy transmission system.

First, the energy emitting coil and receiving coil should form a pair of LC resonators. As for the coils of different sizes and inductance values, the resonant capacitance can be matched via the operating frequency of the oscillating circuit:

\begin{equation*} \textrm {f}=\frac {1}{2\pi \sqrt {LC}}\tag{1}\end{equation*}

Second, the quality factor (Q value) of the coil has a significant impact on the efficiency of energy transmission. The preliminary study on the design of coil parameters shows that there is an optimal choice between Q and f [21]. Namely resonators with coils of different sizes have different optimal operating frequencies. We use MATLAB to calculate the theoretical Q values with the equation (2).

\begin{equation*} Q=\frac {2\pi L\left ({1-\frac {f^{2}}{f^{2}_{self}}}\right)}{R_{dc}\left ({1-\frac {f^{2}}{f^{2}_{h}}}\right)}\tag{2}\end{equation*}

${f^{2}_{self}}$:self-resonance frequency; ${f_{h}}$: the frequency point when the coil power loss is twice of DC power loss; ${R_{dc}}$: DC resistance value

**FIGURE 4.**
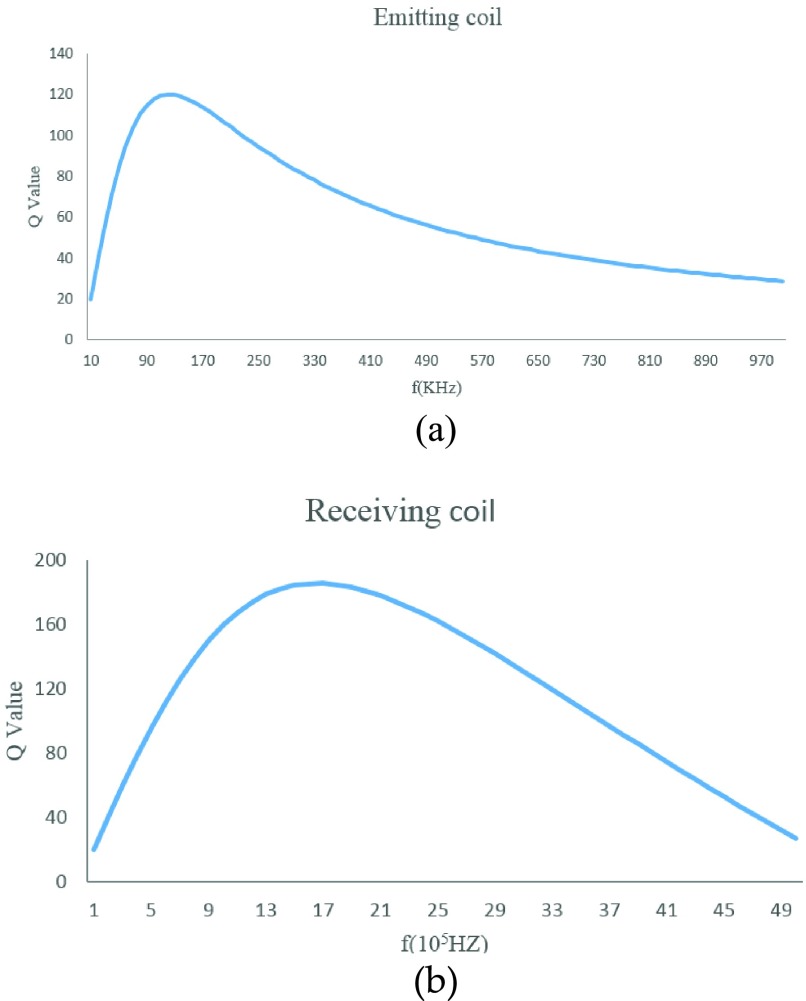
The optimized Q versus operating frequency for each coil. (a) Emitting coil; (b) Receiving coil.

Fig. 4a and Fig. 4b show that our energy emitting coil (loop coil, diameter 8.5cm) gets the maximum Q value at 140KHz, and the best frequency of the receiving coil (rectangular coil, 1.6~1.2cm) is around 2MHz. To meet the requirement of the implantation, the size of the receiving coil is strictly limited so we can only choose one oscillating circuit to work in the best condition. Considering the maximum energy output, the best frequency 140KHz of the emitting coil is chosen as the operating frequency of the wireless energy transmission system.

**FIGURE 5.**
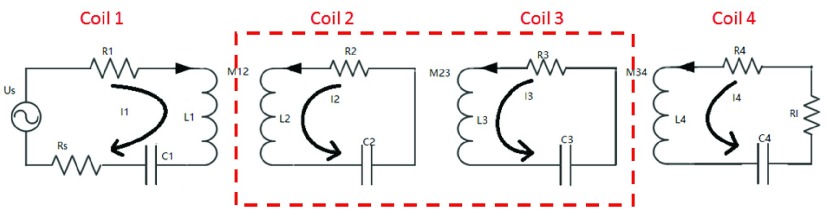
The model of the four-coil magnetic resonance coupling transmission system.

**FIGURE 6.**
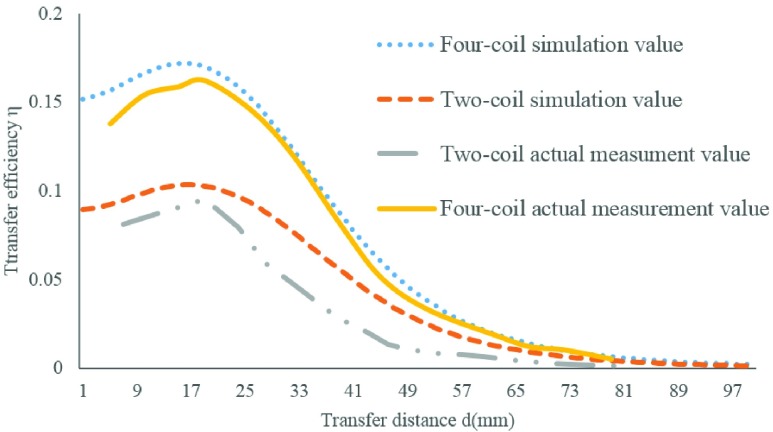
The comparison between the simulation value and the actual measurement value of the two-coil system and the four-coil system.

Third, we used the equation (3) to calculate the efficiency of power transmission. Research suggests that the energy transfer efficiency of the four-coil system is greatly improved compared to the conventional two-coil system [22], [23]. Fig. 5 shows the model of a four-coil wireless energy transmission system; higher efficiency can be achieved because there is no load connection between No.2 and No.3 coil, and longer distance is achieved because of the low consumption on passage. From the contrast of the MATLAB simulation value and the actual measurement value we can see that the maximum transfer efficiency of the four-coil system is 16.20%, while the two-coil system is 9.52%; and the transfer distance extends about 2cm (see Fig. 6).

\begin{equation*} \eta =\frac {P_{\textrm {R}}}{P_{T}}=\frac {V_{R} \times I_{R}}{V_{T} \times I_{T}}\tag{3}\end{equation*}

R: The Received Coil; T: The Transmit Coil

Finally, the resonant coil parameters are designed as shown in Table. 1.

**TABLE 1:** The Coil Parameters of the Wireless Power Transmission System

Parameter	Size	Inductance	Q factor	Resonance cap
Coil 1	Diameter 8.5cm	15uH	117.7	85nF
Coil 2	Diameter 8.5cm	15uH	117.7	85nF
Coil 3	1.2*1.6cm	50uH	21.3	24nF
Coil 4	1.2*1.6cm	50uH	21.3	24nF

**FIGURE 7.**
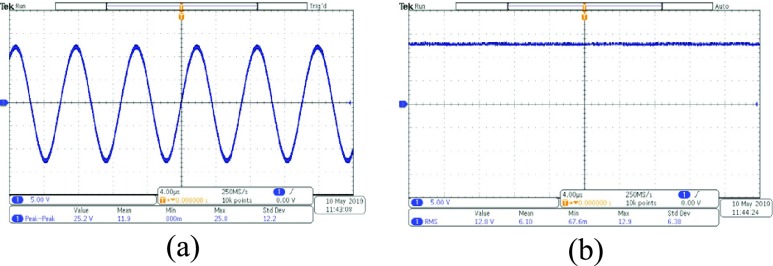
The waveform of voltage before and after the rectifier and filter. (a) The wave form of the voltage before the rectifier (b) The wave form of the voltage after the rectifier and filter.

We used the designed coil to do the experiment of energy transfer. The distance between the coils is 5cm. We get the wave forms of voltage with an oscilloscope (Tektronix MDO3102). The peak-to-peak value of voltage received by the receiving coil is 25.2V (see Fig. 7a). After treatment of rectification and wave filtration, the root-mean-square of voltage is about 12.8V (see Fig. 7b). Then the voltage is converted by a linear voltage regulator to 3.3V which is used as power source of all the systems.

## III. Results

### A. System Validation in Vitro

**FIGURE 8.**
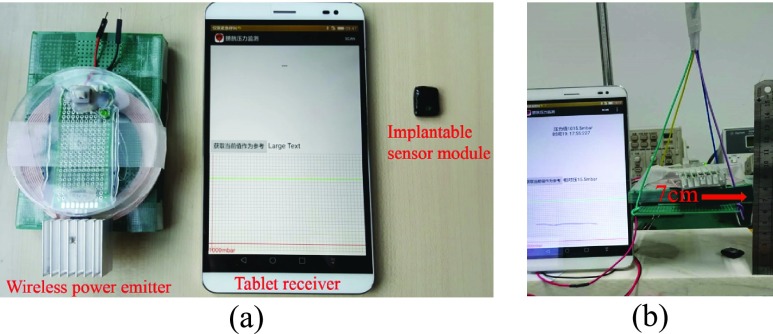
The bladder pressure monitor system. (a) System modules; (b) Working distance test.

Fig. 8a shows the three main physical modules of the bladder pressure monitor system. The system consists of a wireless power emitter, a tablet receiver and an implantable sensor module.

The output power of the wireless power emitter is up to 800mW. The mean power consumption of the implant module is about 2mA. The test result of the maximum working distance shows that the pad receiver can get the pressure data as long as the distance between the wireless power emitter and the implantable sensor module is less than 7cm (see Fig. 8b).

**FIGURE 9.**
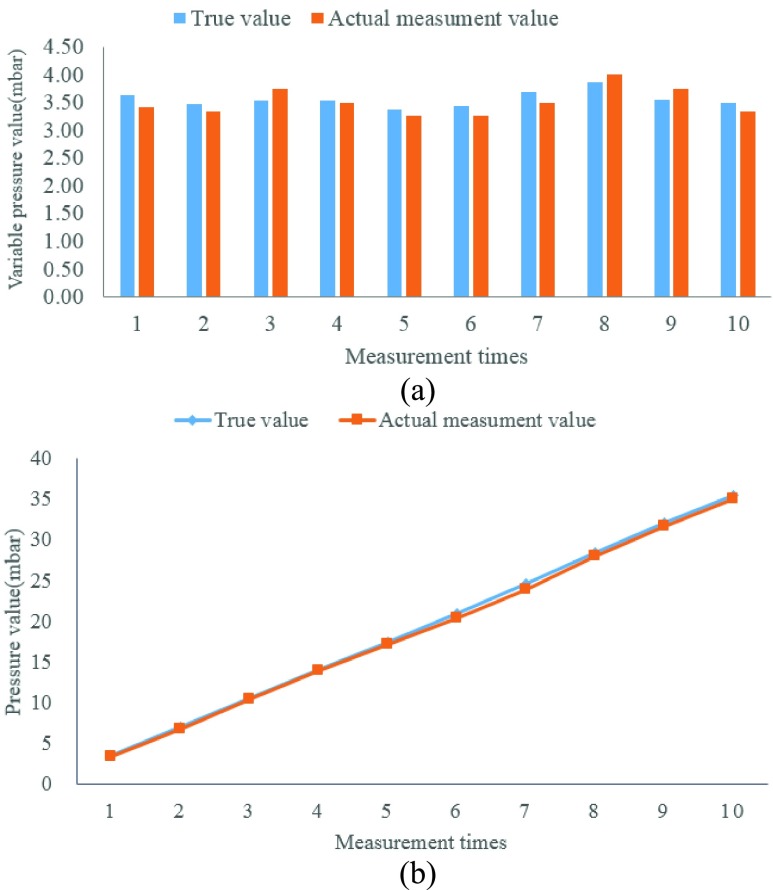
The comparison between the true value and the actual measurement value. (a) Pressure change after adding a small beaker of water. (b) The change in total pressure as the number of measurements increases.

To verify whether the actual measurement accuracy is satisfactory, the implant module was put into a graduated cylinder and water was added by a small beaker. The liquid level increased about 3.5cm after each water flooding and the theoretical pressure value increased about 3.5mbar every time. We recorded the change in pressure (see Fig. 9a) and the total pressure with each addition of a small beaker of water (see Fig. 9b). After that we compared the actual measurement pressure value that was sent by the implantable sensor module with the true calculation value. The experimental result shows that they are basically consistent, and the system error is less than ±0.5mbar. From the datasheet we find C29 sensor is a linear component, and the pressure range of bladder (0–200 mbar) is included in the linear scope so it conforms to the working conditions of pressure measurement.

**FIGURE 10.**
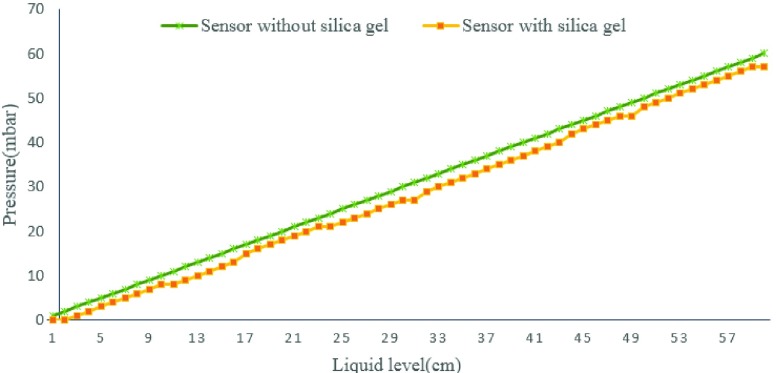
The pressures recorded by different sensors.

In order to avoid urine corresion, we protect some of the implant modules with a layer of silica gel film. To verify the influence of silica gel on the accuracy of pressure value, we recorded the value of pressure before and after wrapping silica gel film by putting the module into a long pipe and adding water to the pipe. The pressure of module with silica gel has a same trend to the module without silica,but there is an error between them(see Fig. 10). The pressure value recorded by sensor with silica gel is 2 mbar lower than the sensor without silica. This error is stable and can be corrected by later mathematical calculation.

### B. Experiment in Vivo

**FIGURE 11.**
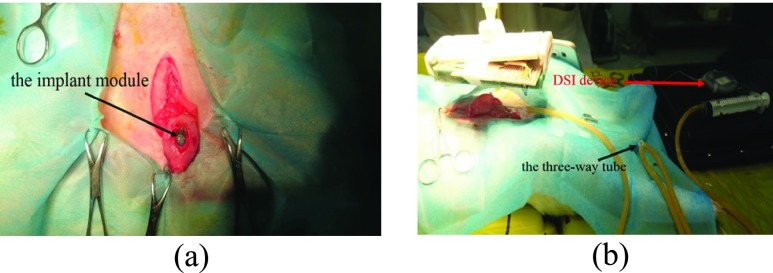
Experimentation. (a) The implant module was placed into the bladder; (b) The three-way tube was used to connect the DSI device, injector and bladder together.

**FIGURE 12.**
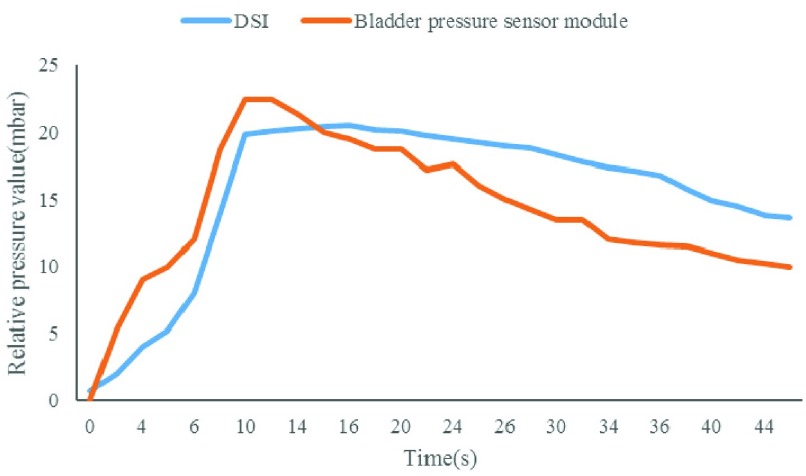
Trend comparison of measurement results of the physiological telemetry system and the bladder pressure sensor module.

The experimental objects of the animal experiments were Japanese big ear rabbits. All animal care and experimental procedures were reviewed and approved by ethics committee of capital medical university. Animals were purpose-bred for research use and procured from certified vendors. Before implantation, the subject was anesthetized (pentobarbital sodium 30mg/kg, ear vein injection). A small incision was made on the bladder to put the sensor module inside (see Fig. 11a). The silicone encapsulated shell of the implant module was sutured with the inner wall of the bladder for fixation before the bladder was sutured. The reference pressure value was detected by a physiological telemetry system (DSI, USA). To conduct pressure equally, a three-way tube was used to connect the sensor probe of the DSI device, the saline injection end and the third end that was connected with the bladder. The wireless energy emission module was then placed in the 7cm range above the subject’ s abdomen; an Android tablet was used to establish the BLE connection and receive the measured pressure data when the implant module began to work (see Fig. 11b). Fig. 12 shows that both the physiological telemetry system and the bladder pressure sensor module could detect the increase of the bladder pressure when saline was injected through the tube, the two pressure curves have the same trend and the measurement values are correlated. SPSS statistical software was used to analyze and find that the measured value of the implantation module over time had a good correlation with the reference pressure (p<0.001, r = 0.815), which indicated that the pressure sensitivity of the device was high, and it could quickly respond to the change of bladder pressure.

To explore the possibility of long-term implantation, ten rabbits (female, 2.3 to 2.6kg or 6–7 months) were randomly selected to conduct an eight-week implant experiment. The reason why the female rabbit was selected for the experiment was its urethra is short and straight.1# and 2# were implanted with the encapsulated implant modules whose sensor windows was protected by a layer of silica gel film to avoid urine corrosion, 3#, 4# and 5# were implanted with unprotected implant modules while the rest were control groups.

**FIGURE 13.**
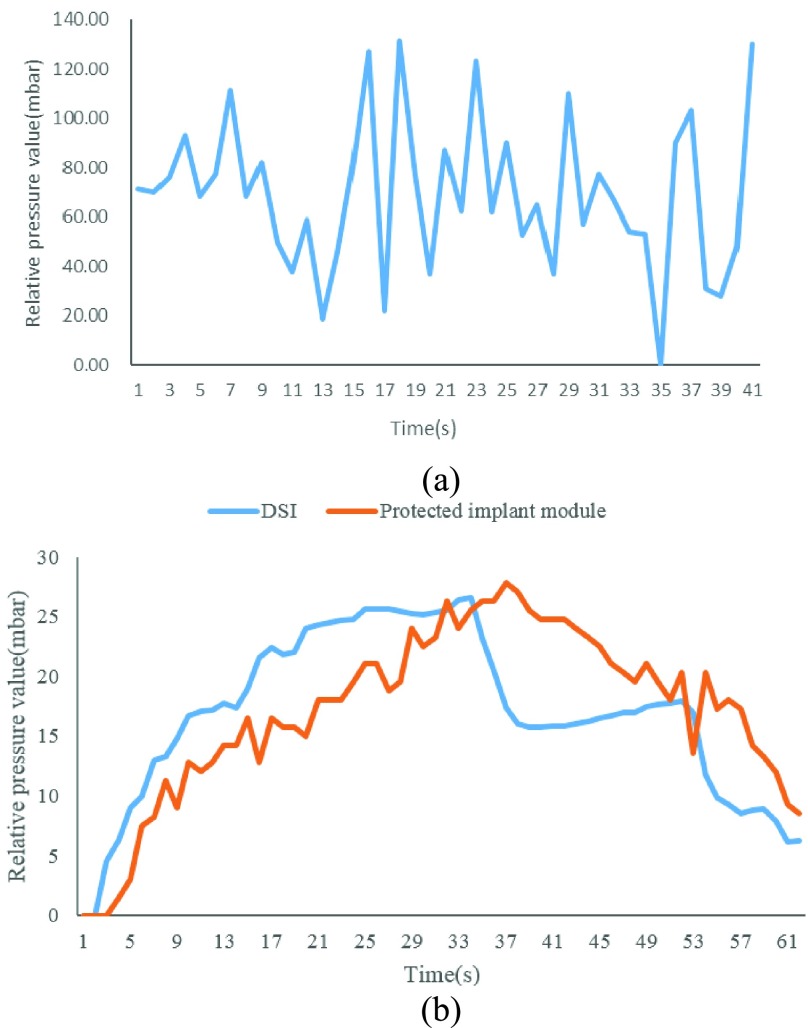
The measurement results of the eight-week implantation experiment. (a) Unprotected implant modules can’t work well anymore; (b)Comparison of the measurement results of the physiological telemetry system and the protected implant module.

The test result shows that the implant modules are still able to be started up by the wireless power emitter after eight weeks. The unprotected modules’ pressure sensor broke (see Fig. 13a), while the protected ones were still able to obtain stable measured data which are consistent with the DSI equipment. The measurement sensitivity decreases due to the silica gel film and mineral encrustation (see Fig. 13b). All the implant modules were separated from the inner wall of the bladder at last. The results proved that it is not advisable to fix the pressure sensor module directly inside the bladder.

**FIGURE 14.**
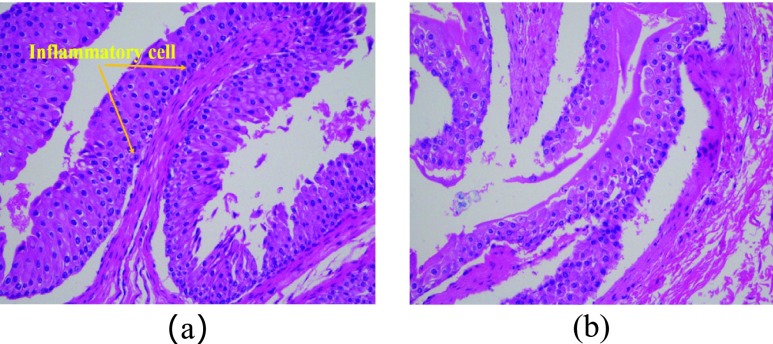
The results of pathological analyses. (a) The experimental group (b) The control group.

After the end of the long-term implantation experiment, bladder tissues were removed from the euthanized rabbits to perform pathological analyses of the tissue sections (see Fig. 14). Twenty tissues were taken from the experimental group and the control group for tissue section analysis and corresponding data statistics. We put two images to compare the difference of bladder between the experimental group (see Fig. 14a) and control group (see Fig. 14b). We measured the bladder wall thickness between the experimental group and the control group and found no significant difference between them (see Table. 2). The pathological analysis result shows that there is no statistical difference of inflammatory response or structural change between the experimental group and the control group, and the higher-grade area of the inflammatory response is mainly in the incision, which means that the implantable sensor module is harmless to the organism.

**TABLE 2:** Pathological Analysis Result

Group	Thickness of bladder(mm) (Mean ± SD)
Experimental	2.21 ± 0.86
Control	2.01 ± 0.67

## IV. Conclusion

The bladder pressure monitor system introduced in this paper has completed design and fabrication, and conducted in vitro experiments and in vivo experiments. The implantable sensor module has a size of 12*17 mm and its average driven current is less than 2mA. With the help of the four-coil wireless energy transmission method, the greatest effective distance is up to 7cm. The measurement result of the sensor is accurate, the data correlates well with the reference device and the trend of the pressure curves is consistent. The pathological analysis result shows that the implantable module with biocompatible encapsulation does not cause any infection, severe inflammation or other negative effects. Therefore, the implantable module is suitable for long-term battery free operation in vivo, and the system can provide bladder pressure feedback to enhance neuro stimulator device therapies and their outcomes.

This system is an important part of bladder function recovery in patients with spinal cord injury. With this system, patients can know their bladder pressure anytime and anywhere and take corresponding measures to avoid problems caused by excessive bladder pressure. Bladder function can be reconstructed or restored in patients with spinal cord injury by combining it with the nervous stimulation system.

Although our system can work without batteries, it still has some improvements to be made, such as analyzing the changes in voltage received at different resonant frequencies. More tests and analysis on magnetic coupling resonance principle are needed to further improve the efficiency and reliability of wireless power supply. Although urine corrosion is avoided by the silicone encapsulation, the performance of the implant module will become worse due to mineral encrustation if it is implanted into the bladder lumen directly, and the fixation method of suturing on the inner wall of the bladder cannot hold for a long time. Hence a more suitable implant position or a surface coating material that can avoid mineral encrustation needs to be found for the sensor module to complete the bladder pressure measurement.
